# Reference Values of Pulse Wave Velocity in Healthy People from an Urban and Rural Argentinean Population

**DOI:** 10.1155/2014/653239

**Published:** 2014-08-24

**Authors:** Alejandro Díaz, Cintia Galli, Matías Tringler, Agustín Ramírez, Edmundo Ignacio Cabrera Fischer

**Affiliations:** ^1^School of Health Sciences, National University of the Center of Buenos Aires Province, 4 de Abril 618, 7000 Tandil, Buenos Aires Province, Argentina; ^2^Favaloro University (AIDUF-CONICET), Buenos Aires, Argentina

## Abstract

In medical practice the reference values of arterial stiffness came from multicenter registries obtained in Asia, USA, Australia and Europe. Pulse wave velocity (PWV) is the gold standard method for arterial stiffness quantification; however, in South America, there are few population-based studies. In this research PWV was measured in healthy asymptomatic and normotensive subjects without history of hypertension in first-degree relatives. Normal PWV and the 95% confidence intervals values were obtained in 780 subjects (39.8 ± 18.5 years) divided into 7 age groups (10–98 years). The mean PWV found was 6.84 m/s ± 1.65. PWV increases linearly with aging with a high degree of correlation (*r* ^2^ = 0.61; *P* < 0.05) with low dispersion in younger subjects. PWV progressively increases 6–8% with each decade of life; this tendency is more pronounced after 50 years. A significant increase of PWV over 50 years was demonstrated. This is the first population-based study from urban and rural people of Argentina that provides normal values of the PWV in healthy, normotensive subjects without family history of hypertension. Moreover, the age dependence of PWV values was confirmed.

## 1. Introduction

The arterial stiffness is an index of vascular health and has been shown to confer additional independent predictive value for adverse cardiovascular outcomes in patients with uncomplicated essential hypertension [1] as well as in the general population [2, 3].

The measurement of carotid to femoral pulse wave velocity (PWV) is considered the gold standard method for arterial stiffness assessment in daily practice because of its easy use, low cost, and high reproducibility [4].

Reference values of PWV come mostly from multicenter registries obtained in Asia [5], USA [6, 7], Australia [8], and Europe [9, 10]. However, several studies in Latin and Hispanic populations have shown significant differences in size, structure, and arterial stiffness of large and small arteries [11–15]. In addition, there are very few population-based studies that evaluate arterial stiffness from populations of South America [16, 17].

At present, there are no reference values of PWV based on healthy normotensive Argentinean population studies involving adolescents, young adults, and older people that take into account factors influencing PWV values such aging [9].

The aims of this work were (a) to establish normality values of carotid-femoral PWV in a large cohort of healthy and normotensive population with no cardiovascular risk factor according to age and (b) to determine the relationship of carotid-femoral PWV with aging and identify the rate of change of this parameter in a healthy population of a community-based study.

## 2. Materials and Methods

### 2.1. Study Population

This project was part of an epidemiological study aimed at determining the prevalence of cardiovascular risk factors in a well-characterized population. The present analysis is a descriptive, observational, and cross-sectional population-based study carried out in healthy people from the population of Tandil city. This project had two phases. The first was focused on evaluating cardiovascular risk factors in the elementary, middle, and high school populations (children and adolescents) [18, 19]. The second phase consisted of blood pressure measurements and cardiovascular risk assessment in the adult population.

Tandil is the main city of the homonymous department, located in Argentina in the southwest of Buenos Aires Province. This city is located 180 meters above sea level and 360 km from Buenos Aires city (37°19′08′′S 9°08′05′′W). The Tandilean population is originated from native inhabitants and a large immigration influx from Italy, Spain, France (Basques), and Denmark. The population is distributed along an urbanized area of 22.07 km^2^ (13.7 mi^2^) surrounded by a suburban area of 30.3 km^2^ (18.8 mi^2^). The latest population data from the National Institute of Statistics and Census reported 123.871 inhabitants in 2010 [20]. All subjects were evaluated in Rodriguez Larreta Hospital and in Tandil Institute of Cardiology. The protocol was approved by the Institutional Ethics and Research Committee, and it was conducted according to the Declaration of Helsinki and the Good Clinical Practice Guidelines. Written consent describing the health examination and type of data collected was obtained from all participants.

### 2.2. Subject's Selection

Between March 2010 and December 2012, a total of 780 consecutive healthy subjects were enrolled. During their routine checkup, we measured the carotid-femoral PWV. Our* normal population* was defined as asymptomatic nonsmoking subjects, having optimal or normal blood pressure values, without diabetes or dyslipidemia and no history of hypertension in first-degree relatives.

### 2.3. Inclusion Criteria

1. Asymptomatic subjects from 10 to 98 years old without history of cardiovascular, pulmonary, and renal disease,
2. serum total cholesterol (TC) levels < 200 mg%,
3. serum triglycerides (TG) levels < 150 mg%,
4. glucose levels < 110 mg%,
5. nonsmoking patients,
6. patient with normotensives first-degree relatives with no history of cardiovascular disease before 65 years of age.

For normal PWV values, the population was categorized according to the age decade (group 1: 10–19 years, group 2: 20–29 years, group 3: 30–39 years, group 4: 40–49 years, group 5: 50–59 years, group 6: 60–69 years, and group 7: ≥70 years old).

### 2.4. Exclusion Criteria

1. Subjects with high blood pressure at the time of the examination,
2. history or symptoms of cardiovascular disease (including arrhythmias),
3. diabetes,
4. serum creatinine levels > 1.5 mg/dL,
5. smokers,
6. lipid profile with one or more of the following conditions: TG ≥ 150 mg/dL, TC ≥ 200 mg/dL,
7. subjects with BMI ≥ 30 kg/m^2^.

### 2.5. Blood Pressure Measurements

Three blood pressure measurements were made, with the patient at rest seated for at least 10 minutes. An individual was defined as being normotensive when presenting with systolic blood pressure (SBP) < 140 mmHg and diastolic blood pressure (DBP) < 90 mmHg. In all cases a digital automatic blood pressure monitor was used (Omron 705IT).

### 2.6. Pulse Wave Velocity Measurement

Pulse wave velocity values were obtained according to the technique previously published and developed in Favaloro University [21, 22]. Briefly, two high-fidelity silicon piezoresistive pressure sensors (Motorola MPX 2050, Motorola Inc., Schaumburg, IL, USA) were connected to an amplifier during data acquisition. The sensors were applied in two different sites of the same arterial pathway. The data was acquired in a computer with specific software that allowed obtaining the time delay between the two instantaneous arterial pulse waves obtained. The software works in a Windows environment performing an online digitized pressure wave acquisition that allows several PWV measurements along a single continuous record, which includes at least 10 cardiac cycles. The same two physicians, one always obtaining the pressure waves and the other operating the computer, performed the data collection in order to avoid operator to operator differences. The quality of the pulse waves was monitored online by the operators and the acquisitions were repeated if necessary. The software was able to calculate the PWV online, taking into account the distance measured between sensors. In this study, the sensors were positioned in (a) the carotid arteries and (b) the femoral arteries, for carotid-femoral PWV evaluation.

Measurements were performed in a quiet room with stable room temperature with a patient in a supine position after 10 min of rest. Measurements were obtained by duplicate [4]. The mean and the standard deviation of these measurements were always calculated and considered as the PWV value for each patient. In order to ensure a reliable measurement, special care was taken in monitoring that the standard deviation of measurements was less than 10%.

In accordance with international recommendations, the corrected PWV was calculated by multiplying by 0.8 [23], as suggested by the European recommendations [4]. In this research, 10 meters/second was considered as limit value for normal value.

### 2.7. Body Mass Index Calculation

The weight was measured with the patients in the orthostatic position, with the arms extended along the body, being barefoot, and wearing light clothes. A stadiometer with a precision of 0.1 cm was used to measure height, with the participants standing barefoot. The body mass index (BMI) was calculated as weight in kilograms divided by the square of the height in square meters (kg/m^2^). Normal weight was considered when BMI was in between 18.5 and 24.9 kgm^2^. Overweight was defined when BMI was >25 and <29.9 and, finally, obesity was diagnosed when BMI was higher than 30 kg/m^2^.

### 2.8. Laboratory Measurements

Venous blood samples were obtained in the forearm by standard techniques and processed for determination of serum triglycerides, total cholesterol, glucose, and creatinine.

### 2.9. Statistical Analysis

Measured and calculated values were expressed as mean value ± SD. A *P* < 0.05 was considered statistically significant. Differences among groups were tested by means of ANOVA and Bonferroni's posttest. Statistical analyses were done using Statistical Package for the Social Sciences 19.0 (Chicago, IL, USA).

## 3. Results

Demographic characteristics of the seven hundred eighty healthy subjects included in this research (age 39.8 ± 18.5 years, range 10–98 years) are summarized in Table 1. The mean PWV found was 6.84 m/s ± 1.65 (range: 3.12–13.4).  Table 2 shows the mean PWV, the range, and the 95% confidence intervals in normal subjects divided into 7 age groups. Note that the standard deviation of the first four age groups (10 to 49 years) is smaller than that of the last three age groups (50 to 98 years) indicating an increase in the scatter of the PWV with the aging process. Similarly in subjects older than 70 years minimum and maximum values of the 95% confidence interval show a greater dispersion than that observed in younger healthy participants.

**Figure 1:**
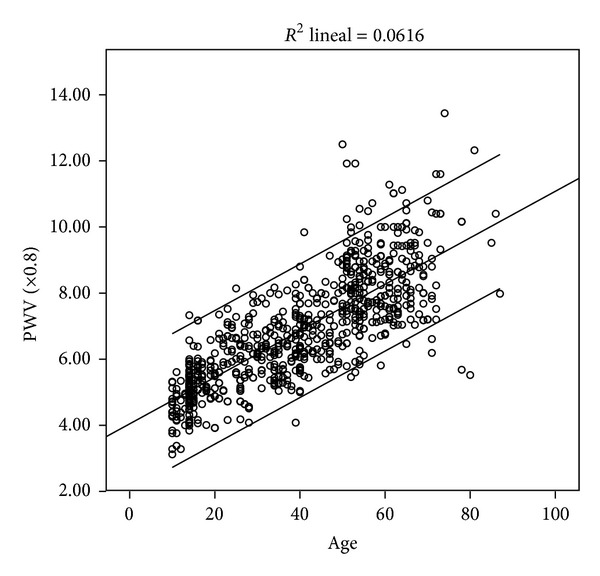
Scatter graph showing the relationship between mean PWV (mean and CI 95%) and age in the study population (*n* = 780).

**Figure 2:**
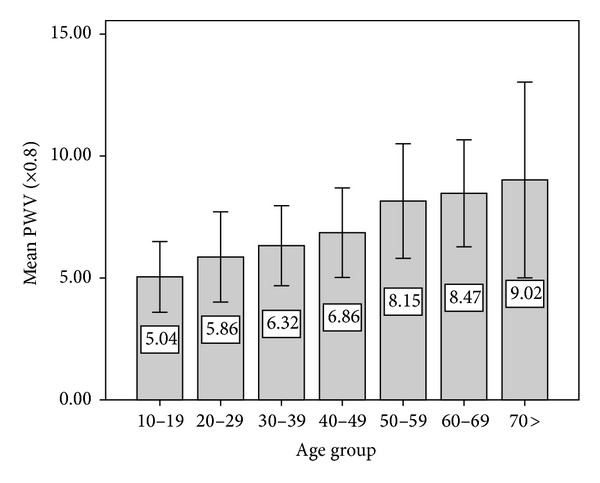
Evolution of the mean values of the pulse wave velocity (mean ± SD) according to age groups.

Figure 1 shows that the PWV increases linearly with aging with a high degree of correlation (*r* ^2^ = 0.61; *P* < 0.05) with less dispersion in younger subjects. In our population, PWV progressively increases averaging 6–8% with each decade of life, and this tendency is more pronounced after 50 years in which the average PWV increased by 18%. See Figure 2 and Table 2.

No differences in PWV values were found between men and women (6.81 m/s versus 6.89 m/s, resp.). This lack of gender statistical difference was also observed in all age groups.

**Figure 3:**
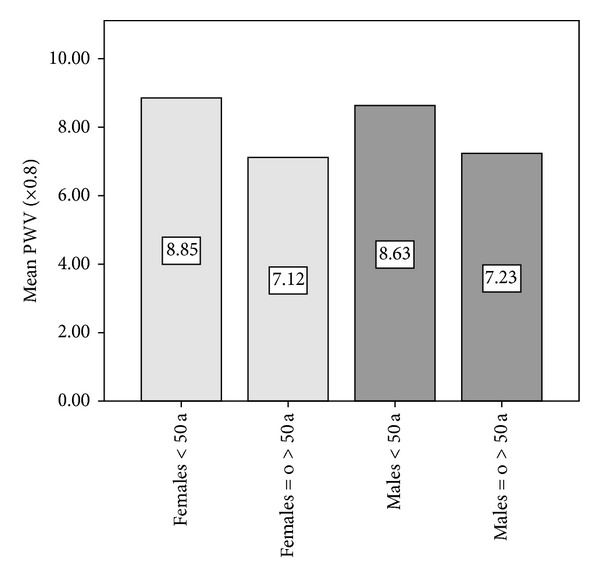
Mean values of pulse wave velocity (PWV: in meters per second) showing significant differences between young subjects (≤50 years) and subjects > 50 years included in the study. There were no significant differences of PWV in relation to gender for the same age group.

PWV values were higher in subjects over 50 years (8.35 versus 5.92 m/s). This difference remained statistically significant in both men (8.52 ± 1.39 versus 5.86 ± 1.17 m/s) and women (8.20 ± 1.13 versus 6.03 ± 1 m/s).  Figure 3 shows that the differences are mainly determined by the aging and are significant between young and elderly subjects regardless of gender (*P* < 0.05).

## 4. Discussion

The measurement of PWV is a well-known method for the quantification noninvasive arterial stiffness and is currently considered the gold standard of arterial stiffness due to its simplicity, accuracy, reproducibility, and predictive value [24, 25].

Most studies that establish reference values of PWV include data obtained from retrospective analysis of patients evaluated in different specialized centers. The mentioned reports includes several selection bias that difficult comparative studies with other patient populations. Despite the recognized value of PWV for predicting cardiovascular risk, in South America there is a scarcity of reference values. Moreover, only the Republic of Uruguay has reference values of PWV based on urban population [16].

The present research clearly shows the normal values of PWV with the corresponding confidence interval of 95% for each age group providing relevant clinical information in terms of aortic stiffness. Since PWV is an age dependent parameter, the clinician needs to know the mean value and calculated dispersion for each decade of human life in order to orientate both diagnosis and preventive strategies.

Our work has several relevant aspects to comment on.

First, this work represents the first Argentine record based on urban and rural population that determines normal values of PWV in a large number of normotensive and healthy subjects free of family history of hypertension. On the other hand, the results of this based population study could only be extrapolated to those communities with similar demographic characteristics. It should be noted that several demographic and sociocultural aspects of the population of the city of Tandil have similarities with the general population of Argentina and South America (Table 3) [26]. Indeed, the percentage of women and the elderly as well as literacy rates, infant mortality, unemployment, and incomes (gross domestic product) is comparable to those of the general population of Argentina and several countries of Latin America. Moreover, the prevalence of hypertension in Tandil city and in rural areas is comparable to that reported in population-based epidemiologic studies of our country in the last two decades [27, 28] and similar to that reported in other urban and rural populations in Latin America and the Caribbean region [29].

Second, the present research has several peculiarities when compared to other international databases. One of them is similar number of subjects but covering a wider range of ages (10 to 98 years). Farro et al. [16] reported the reference values of PWV in 429 subjects selected from a hospital population from urban Uruguay. They used a methodology similar to ours but considered six age groups and they analyzed subjects older than 60 years as a single group. In addition, the number of individuals in each group is less than our work. On the other hand, the European Registry “Arterial Stiffness Collaboration” [9] reported normal values of PWV based on 1455 records. These normal values emerged from a retrospective analysis of PWV obtained with different methodologies in 13 European centers of high complexity. This registry considered subjects less than 30 years as a single group. Moreover, this record included several methods of measurement of PWV and required validation between different methodologies with international comparative study subjects to verify measurement accuracy. In the research here reported, all data collection was obtained by only one research group, always using the same technology. This methodological aspect allows us to avoid interobserver group differences.

The third important aspect is the different rate of increase in PWV observed across different age groups. In our population, there is a deterioration of arterial elasticity associated directly with age across all the ages studied; however, in agreement with previous reports [8], PWV presents a different behavior before and after the age of fifty (Figure 3).

Fourth, with respect to gender difference in arterial stiffness, the authors are aware of the controversial reports about this interesting issue [9, 30]. In our study, there were no statistically significant differences in PWV values linked to gender. This finding is in concordance with reported large population-based studies in which the gender difference in PWV was absent or without clinical significance (<0.1 m/s) [8, 13, 31]. In the framework of the Anglo Cardiff Collaborative Study and Multiethnic Study of Atherosclerosis, it was demonstrated that gender might not directly influence arterial stiffening in healthy normotensive individuals [8].

Fifth, in our study, we included an important aspect to define “normal values of PWV” as it is the absence of first-degree relatives with a history of hypertension, coronary heart disease, or sudden death before age 65 [32]. Several studies have shown a significant hereditary burden on several indexes of arterial function independently of blood pressure values and there is an association between PWV and genetic polymorphisms [6, 33, 34]. Both associations justify analyzing a redefinition of the inclusion criteria to define a population as the reference standard.

With respect to the methodological approach used in this work, the authors take into account previous reports in which comparative studies with invasive methods show that the use of direct measurement of carotid-femoral distance results in an overestimation of up to 25.4% of the distance traveled by the pulse wave and consequently results in overestimation of PWV values in 2-3 m/s [4, 23]. In consequence, we used the corrected carotid-femoral distance (x 0.8) which is the distance that has proven to be the best correlate with the “real” distance traveled by the pulse wave [23].

Finally, the relevance of routine PWV measurements under standardized conditions was emphasized in the last expert consensus on aortic stiffness measurement [4]. Consequently, the availability of PWV values that characterized each decade of the human life in healthy person acquires great relevance in order to establish the degree of functional impairment in different pathological states.

## 5. Conclusion

This research is the first population-based study of an urban and rural population in Latin America that provides normal values of the pulse wave velocity in healthy, normotensive subjects without family history of hypertension. In the analyzed sample, PWV values show a direct association with the aging process. Our data provide relevant clinical information to daily clinical practice setting with PWV cut-off values for each age group with the CI 95%. Moreover, a significant increase in the PWV growth rate after the fifth age decade was confirmed. This supports the idea for an increase in the cardiovascular risk accompanying the ageing process.

## Figures and Tables

**Table 1:** Description of clinical and haemodynamic variables of the examined population.

Variable	Normal population (*n* = 780)
Age (years)	39.8 ± 18.5 (range: 10–87)
Gender (male/female)	414/366 (53.1%/46.9%)
Weight (kg)	64 ± 14.4
Height (m)	1.66 ± 0.11
BMI (kg/m^2^)	24.12 ± 3.84
Waist (cm)	87.7 ± 14.4
SBP (mmHg)	121.03 ± 11.64 (range: 90–139)
DBP (mmHg)	74.84 ± 8.65 (range: 50–89)
PP (mmHg)	46.19 ± 8.48 (range: 20–70)
MAP (mmHg)	90.23 ± 8.89 (range: 66.7–105.7)
Total blood cholesterol (mg/dL)	168.5 ± 22.6 (range: 110–197)
Serum triglycerides (mg/dL)	125 ± 20.5 (range: 78–148)
Glycaemia (mg/dL)	82.4 ± 9.5 (range: 56–103)

BMI: body mass index; SBP: systolic blood pressure; DBP: diastolic blood pressure; PP: pulse pressure; MAP: mean arterial pressure.

**Table 2:** Carotid-femoral PWV for each age group in the examined population.

Age group (years)	*n*	Mean PWV (m/s)	SD	95% CI Lower-upper limit	Range
10–19	156	5.04	0.72	4.92–5.15	3.12–7.33
20–29	110	5.86	0.92	5.68–6.03	3.92–8.14
30–39	109	6.32	0.82	6.16–6.47	4.08–8.26
40–49	108	6.85	0.91	6.68–7.03	5.0–9.84
50–59	164	8.15	1.17	7.97–8.33	5.46–12.5
60–69	103	8.47	1.09	8.25–8.68	6.46–11.2
>70	30	9.01	2.00	8.27–9.76	5.52–13.4

Total	780	6.84	1.65	6.73–6.96	3.12–13.4

PWV: pulse wave velocity, CI: confidence intervals.

**Table 3:** Socioeconomic indicators of Tandil city and Latin American countries (year 2010).

City/country	Population aged ≥ 65 (% of total)	Population, female (% of total)	Literacy rate, adult total (% of people ≥ 15 years)	Mortality rate, infant (per 1,000 live births)	GDP per capita (current US$)	Unemployment, male (% of male labor force)
Tandil	13	51.6	99	12	11.0	7.4
Argentina	11	51.1	98	13	9.1	7.8
Bolivia	5	50.1	91	41	2.0	
Brazil	7	50.8	90	15	11.0	6.1
Chile	9	50.6	99	8	12.6	7.2
Colombia	6	50.8	93	16	6.2	9.1
Ecuador	6	49.9	92	20	4.0	5.2
Paraguay	5	49.5	94	20	2.8	4.4
Peru	6	49.9		15	5.3	4.4
Uruguay	14	51.7	98	9	11.7	
Venezuela	6	49.8	96	13	13.7	7.2

GDP: gross domestic product. GDP per capita (current US$): GDP per capita is gross domestic product divided by midyear population.
