# Corrigendum to “Reference Values of Pulse Wave Velocity in Healthy People from an Urban and Rural Argentinean Population”

**DOI:** 10.1155/2015/983928

**Published:** 2015-03-29

**Authors:** Alejandro Díaz, Cintia Galli, Matías Tringler, Agustín Ramírez, Edmundo Ignacio Cabrera Fischer

**Affiliations:** ^1^School of Health Sciences, National University of the Center of Buenos Aires Province, 4 de Abril 618, 7000 Tandil, Buenos Aires Province, Argentina; ^2^Favaloro University (AIDUF-CONICET), Buenos Aires, Argentina

In the paper titled “Reference Values of Pulse Wave Velocity in Healthy People from an Urban and Rural Argentinean Population” there was an error in Figure 1. The correct value of *R* ^2^ is =0.616. In this erratum we report corrected Figure 1 where the mentioned mistake has been replaced with the corrected value.

In the paper entitled “Reference Values of Pulse Wave Velocity in Healthy People from an Urban and Rural Argentinean Population” a mistake was introduced in Figure 3. In this erratum we report modified Figure 3.

## Figures and Tables

**Figure 1:**
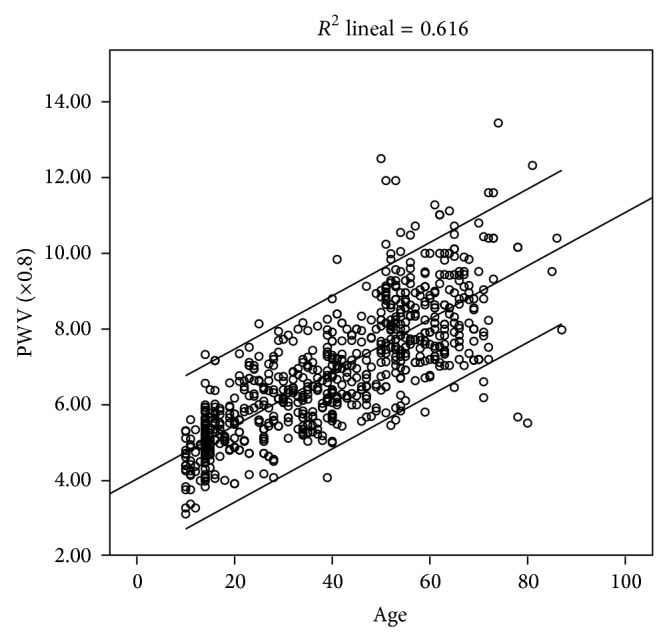
Scatter graph showing the relationship between mean PWV (mean and CI 95%) and age in the study population (*n* = 780).

**Figure 3:**
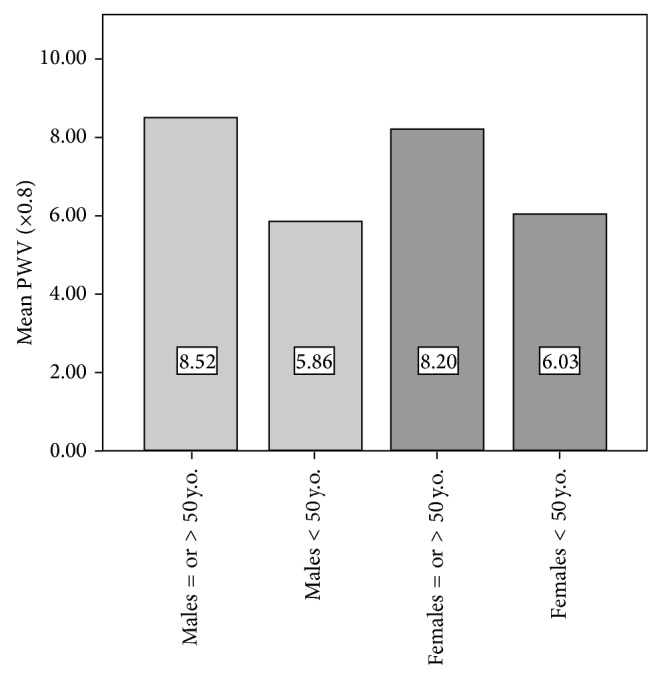
Mean values of pulse wave velocity (PWV: in meters per second) showing significant differences between young subjects (≤50 years) and subjects > 50 years included in the study. There were no significant differences of PWV in relation to gender for the same age group.

